# Glucocorticoid Treatment Strategies in Liver Failure

**DOI:** 10.3389/fimmu.2022.846091

**Published:** 2022-03-16

**Authors:** Chao Ye, Wenyuan Li, Lei Li, Kaiguang Zhang

**Affiliations:** ^1^ Department of Gastroenterology, The First Affiliated Hospital of University of Science and Technology of China (USTC), Division of Life Sciences and Medicine, University of Science and Technology of China, Hefei, China; ^2^ Department of Infectious Diseases, The First Affiliated Hospital of University of Science and Technology of China (USTC), Division of Life Sciences and Medicine, University of Science and Technology of China, Hefei, China

**Keywords:** liver failure, glucocorticoids (GCs), inflammation suppression, immunosuppression, strategies

## Abstract

Liver failure is characterized by serious liver decompensation and high mortality. The activation of systemic immune responses and systemic inflammation are widely accepted as the core pathogenesis of liver failure. Glucocorticoids (GCs) are most regularly utilized to suppress excessive inflammatory reactions and immunological responses. GCs have been used in the clinical treatment of liver failure for nearly 60 years. While there has been no unanimity on the feasibility and application of GC treatment in liver failure until recently. The most recent trials have produced conflicting results when it comes to the dose and time for GC therapy of different etiology of liver failure. Our review outlines the issues and options in managing GC treatment in liver failure based on an investigation of the molecular mechanism that GC may give in the treatment.

## 1 Introduction

Liver failure (LF) is a life-threatening syndrome defined as the acute decompensation of liver function with varied etiology and multiple organ dysfunctions (1, 2). In China, viral hepatitis is the leading cause of liver failure, followed by alcohol and toxic drugs. Hepatitis B virus (HBV) related acute-on-chronic liver failure (ACLF) is the most common type of end-stage liver disease in chronic HBV infection patients, characterized by rapid deterioration, with muti-organ failure and high short-term mortality (3). At present, there is still no specific treatment of LF. Currently treatment is mostly based on comprehensive medical care, artificial liver support systems (ALSSs), and liver transplantation (LT). LT is an effective therapy even in patients at advanced stages, nevertheless, LT is limited by the availability of donor organs and the high medical cost, the mortality rate of LF remains high (4, 5).

Activation of immune response and systemic inflammation are considered as the key role of LF, glucocorticoids (GCs) have been used in the clinical treatment of LF for many years with the function that can rapidly suppress excessive inflammatory reactions and immune response. However, their usage has been contentious. For over half a century, there are numerous studies have been published (6–9). Although some clinical and experimental research are currently being conducted to determine the efficacy of GC treatment of LF, countries and organizations have yet to reach a consensus (10–15).

This study covers advances on the mechanism, value, existing difficulties, and application tactics of GC application in different etiology LF to identify ideas for further research in related domains and to provide assistance for the clinical management of LF.

## 2 The Mechanism of GC Treatment in Liver Failure

### 2.1 The Core Pathogenesis of Liver Failure

Liver acts as an immune organ and plays a key role in innate immune defenses against pathogens (16–18). LF has the features of systemic inflammation, cellular immune depression, and progression to multiple organ dysfunction. Activation of systemic immune responses should be considered playing a significant role in the pathogenesis and prognosis of LF (19, 20). Cytokines also play a pivotal role in LF pathophysiology including hepatocellular death, extrahepatic complications, and hepatocyte regeneration. And cytokines mediated liver injuries are tightly associated with hepatocyte proliferation and regeneration (21, 22). Suppressor of cytokine signaling (SOCS) family, signal transducer and activator of transcription (STAT) and nuclear factor kB (NF-kB)-mediated pathways have been shown closely linked with liver injury (23, 24).

Patients with ALF and ACLF display evidence of a pro-inflammatory state with local liver inflammation, features of systemic inflammatory response syndrome (SIRS) and vascular endothelial dysfunction that drive progression to multi-organ failure (25). The sooner SIRS emerges, the worse the prognosis (26). “Endotoxin-macrophage-cytokine storm” is the core pathogenesis of liver failure (27). The immunological balance is disrupted in the latter stages of liver failure, resulting in “immune paralysis” and a reduction in the total number and activity of peripheral blood lymphocytes, both of which aided in the progression and exacerbation of LF (28–30).

The “first hit” in the “three hits hypothesis” is the initial immunological insult to the liver produced by viruses, medications, and other factors, which immediately leads to the degeneration and necrosis of hepatocytes. The loss of hepatic sinusoids, microvascular embolism, and microcirculation disturbances result in ischemia and hypoxia of liver tissue, as well as additional reperfusion damage, resulting in the “second hit”. The liver’s detoxifying and endotoxin-scavenging abilities were reduced by the first two strikes, resulting in intestinal endotoxin-induced endotoxemia, which released a significant number of inflammatory factors such as IL-6 and tumor necrosis factor α (TNF-α), culminating in the “third hit” (31–33). GCs can impede macrophage phagocytosis and antigen treatment, as well as reduce the generation of inflammatory cytokines, since they are the most often utilized anti-inflammatory and immunosuppressive medicines. As a result, there is a theoretical foundation for using GCs to treat liver failure.

### 2.2 Immune Response Inhibition and Anti-Inflammatory Mechanisms of GCs

GCs can swiftly suppress excessive immune response and inflammatory reaction. In addition to inhibiting cytotoxic liver damage, GC intervention in LF can also control humoral immunity. GCs can influence the fraction of CD4+ lymphocyte subsets that are distributed, raise the proportion of Treg cells, and boost the immunomodulatory activity of Treg cells, all of which contribute to increased negative inflammatory control (34). Furthermore, GCs can directly decrease CD8+ cell immunological activity and diminish cytotoxicity. ICAM-1 (intercellularcelladhesionmolecule-1, ICAM-1) is a part of the immunoglobulin superfamily which found on the cell membrane of hepatocytes and many other cells. ICAM-1-mediated cell adhesion is critical for cytotoxic T lymphocyte (CTL) attachment to hepatocytes and can improve CTL assault on target cells. At the receptor level, GCs can block the production of ICAM-1, effectively preventing CD8+ lymphocytes from attacking hepatocytes. Furthermore, GCs can minimize the liver tissue damage induced by T/NKT cell infiltration by inhibiting the killing impact of T/NKT cells (35, 36).

GCs can induce apoptosis of inflammatory cells and inhibit antigen presentation as well as the generation and release of proinflammatory cytokines including IL-1, IL-6, TNF-α, and IL-17 (37, 38). GCs can also boost the synthesis of the anti-inflammatory cytokine IL-10 and improve the negative control of inflammatory factor storms at the same time (37). Furthermore, through modulating the immunological signal transduction pathway, GCs can decrease the inflammatory response. Important negative cytokine regulatory factors include suppressor of cytokine signaling 1 (SOCS1), suppressor of cytokine signaling 2 (SOCS2), and interleukin-1 receptor-associated kinase M (IRAK-M) (39). GCs can improve the inhibitory impact of SOCS1 and SOCS2 on the JAK/STAT inflammatory signaling pathway, as well as the inhibitory effect of IRAK-M on the Toll-like receptor 4 (TLR4) inflammatory signaling pathway (40, 41). Nucleotide-binding oligomerisation domain-like receptors (NLRs) Family Pyrin Domain Containing 3(NLRP3) is related to innate immunity and can produce proinflammatory cytokines *via* caspase-1 (42). NLRP3 has been found increased in HBV-related ACLF patients and downregulated by GCs in surviving patients (43). GCs can also cause lymphocytes to migrate out of blood vessels, reducing the number of lymphocytes in blood vessels. It may also enhance the local microcirculation of the liver, as well as lessen the disturbances of ischemia, hypoxia, and reperfusion of hepatocytes.

### 2.3 GCs Can Enhance the Protective Effect of Hepatocytes

By inhibiting caspase-8 activation and the mitochondria-dependent apoptosis pathway, dexamethasone (DEX) pretreatment protected hepatocytes from TNF-α, plus actinomycin D (ActD)-induced apoptosis (44). Considering that GCs are thought to have a significant stabilizing impact on cell membranes, they can prevent hepatocyte disintegration and necrosis, slowing the course of liver damage.

## 3 GC Therapy in Different Etiology of Liver Failure

### 3.1 HBV Related ACLF

According to the evidence shows that HBV mainly causes liver damage through cytotoxic T-lymphocyte-mediated cytolytic pathways in HBV-infected hepatocytes (45, 46), using GCs to treat severe hepatitis B infections is appropriate because of the particular effects of GCs to inhibit immune responses and prevent cytolysis in infecting hepatocytes (47). Multiple studies suggest that using GCs in the early period of severe hepatitis can help prevent liver cells necrosis and afford a possibility of liver regeneration (48–50) but might enhance HBV replication (51), and lead to LF (12, 52–54). Thus, GCs have not been widely used for the treatment of severe hepatitis B in clinic. However, in recent years, due to the new generation of nucleoside analogs (NA), using GCs to treat HBV related LF has become much safer (55–57).

Excessive systemic inflammation and susceptibility to infection are two pathophysiological characteristics of ACLF. Immunotherapies, such as glucocorticoids are effective on ACLF. Some studies have reported that GC treatment improve the survival rate of the patients with HBV-ACLF. A prospective multi-center clinical trial totally included 171 HBV-ACLF patients, 83 patients treated with methylprednisolone [1.5 mg/kg/day (day 1-3), 1 mg/kg/day (day 4-5), and 0.5 mg/kg/day (day 6-7)] for 7 days, the results showed methylprednisolone treatment can increase the 6-month survival rate of HBV-ACLF patients (27). And there is a retrospective study included 349 patients with HBV-ACLF in 2021. 155 patients used methylprednisolone or prednisone. The results showed that GC treatment could not improve the liver function of ACLF patients but might reduce their 28 days mortality rate (58). Similar results were also demonstrated by Zhao et al (59). No matter the patients used antivirus or not. The explanation for this might be that infectious complications are both the primary cause of ACLF and the leading cause of mortality from ACLF, these patients are susceptible immune paresis (60). Thus even though GC therapy did not improve liver function or short-term health, it may be required in critical patients. Nevertheless, this effect has not been validated by others. A retrospective, controlled trial with 31 HBV-related ACLF patients under dexamethasone injection for three times and followed up for 12 weeks, the results showed that dexamethasone cannot improve liver functions and 12-week survival rates of patients with HBV-related ACLF (61). A Ten-year cohort study in a University Hospital in East China also showed that steroid treatment did not improve transplant free survival in ACLF patients precipitated by hepatitis B (15).

The timing of GCs treatment in HBV-related ACLF is very important. Zhang et al. found that dexamethasone (10 mg/day, i.v.) for 5 days based on lamivudine (LMV) treatment is effective in improving the liver function and survival rate of patients with pre-ACLF (62). Another study included 87 patients with early-stage HBV-related subacute liver failure, 43 patients in the control group received LMV and routine integrated treatment, and those in the treatment group were given additional short-term low-dose glucocorticoid treatment. The results showed that GC treatment can improve survival rate and shorten the mean hospital stay of patients with HBV-related early-stage subacute liver failure patients (63). Thus, GCs should theoretically be able to control excessive systemic inflammation and hepatic inflammation in the early stages of ACLF, whereas they aggravate immune paralysis in the late stages. And another noteworthy issue is that nucleoside analog should be used as a basic treatment in HBV-ACLF patients. Overall, low dose, short term GC treatment combined with NA in the early stage of HBV-ACLF patients is safe and effective.

### 3.2 HBV Related ALF

Nearly half a century ago, researchers used double-blind, randomized trials of methylprednisolone(38-48mg/day) vs. placebo in severe viral hepatitis and showed the conclusion that methylprednisolone does not enhance survival in patients with severe viral hepatitis (6, 7). In 2006, Kotoh et al. used a high-dose methylprednisolone (1000 mg/day for 3 continuous days) to treat patients with severe acute hepatic failure and found methylprednisolone might effectively prevent the progression of severe acute hepatic failure (64). Fujiwara et al. used 1000 mg of methylprednisolone daily for 3 days followed by the reduced doses according to the treatment response in the early stage of viral acute liver failure, which indicated an effective suppressing of hepatocytes destruction and a slightly higher survival rate (65). And high dose of GCs treated in ALF did not significantly increase the incidence of infection (66). Then *Fujiwara* reported combination therapy with GCs and NA for HBV-ALF induces the rapid resolution of inflammation leading to a rapid recovery of the liver function. When it is administered at a sufficiently early stage, it would have a survival benefit and prevent persistent infection (67). In a whole, when treated in ALF, high dose GCs might be more effective, the early stage of the ascending period would be the best timing, and NA is also very important in HBV related ALF.

### 3.3 AIH Induced Liver Failure

Autoimmune hepatitis (AIH) is an immune-mediated necroinflammatory disease of the liver parenchyma. Although AIH is linked with minor symptoms in most patients, it can also be associated with severe symptoms and develop to ALF, or ACLF. Acute severe autoimmune hepatitis (AS-AIH) is a relatively rare cause of ALF, which is often neglected and delayed in treatment. The standard paradigm of management in acute AIH involved corticosteroid therapy. This can achieve a remission in more than 80% (68). However, GCs use in AIH-induced LF remains controversial. A single-center French study in 2007 looked at the role of GSs in patients with a severe presentation of AIH. They found that GC therapy is of little benefit in severe and fulminant forms of AIH; It may increase the risk of septic complications and should not delay liver transplantation (LT) (69). A retrospective analysis of patients with autoimmune, indeterminate, and drug-induced ALF included 66 patients with AIH. The study compared 25 patients who were given prednis(ol)one (median dosage 60 mg/day) with 41 patients who were not. GCs did not increase overall or spontaneous (without-LT) survival in autoimmune ALF. Furthermore, GCs use was linked to an increased mortality in the group of patients with the highest MELD scores (70). While there are also researchers suggested GCs should be considered as soon as possible in AS-AIH patients (71). Recently, a retrospectively study enrolled 32 patients with AIH-induced ALF compared with 93 age- and sex-matched patients with chronic AIH (cAIH), the patients received prednis(ol)one with an average dose of 153.9 mg daily for the first group and from 61.8 mg daily for the second group. GCs therapy was not associated with high mortality or sepsis in AIH-induced ALF and suggested that GCs treatment of AIH-mediated ALF may improve the outcome (72). Another study included 128 AS-AIH patients, 115 (90%) were treated with GCs within a median of 6 (2–10) days of their admission to hospital. Seventy-eight patients (73%) received prednis(ol)one with a dosage of 1 mg/kg/d while 37 (27%) received 0.5 mg/kg/d. Thirteen patients (10%) did not receive GCs therapy, the results showed that non-treated patients were more seriously ill than treated patients (73). Zachou et al. present an open, real-world observational study included 34 AS-AIH patients were treated with either 1g methylprednisolone for 3 consecutive days followed by intravenous prednisolone (1mg/kg/day) or prednisolone (1.5mg/kg/day) from the beginning. And indicated that high-dose intravenous GCs in original AS-AIH seems safe and efficient as it prevents disease deterioration and the need of liver transplantation (74). A recent Asian-Pacific study included 82 patients with AIH induced ACLF. A survival benefit was demonstrated in those who received GCs. Moreover, patients with high MELD scores and encephalopathy had unfavorable responses to GCs (75). In general, GC treatment is effective in both ALF and ACLF induced by AIH. High doses are also relatively safe. GCs should be used as early as possible, with an increased risk if MELD score is very high or encephalopathy present.

### 3.4 Drug Induced Liver Failure

Drug-induced liver injury (DILI) is a liver toxicity induced by drugs or their metabolites. Patients with DILI may present with various clinical manifestations, ranging from abnormal liver function test results but without symptoms to ALF (76). For drug-induced ALF, there are two primary therapeutic options: a) fast depuration of the body from the toxic chemical to prevent additional aggressiveness before the agent reaches the liver; and b) administration of an antidote to prevent and/or stop the aggression once the toxin reaches the liver. The newest EASL clinical practice guidelines suggested that GCs are usually given when all else fails to produce results (77). In early trials, GC therapy for all kinds of ALF demonstrated limited benefits (70, 78). A single-centre retrospective study used two kinds of GCs administration methods (Methylprednisolone, range 60-120 mg/day or prednisone, range 40-60 mg/day for 3-5 days and then prednisone 20 mg/day and 5-10 mg weekly reduction) or (Methylprednisolone, range 60-120 mg/day for 3-5days) to treat severe drug-induced liver injury (DILI) patients. The results showed that short-term use of GCs can improve the liver injury and patient survival of severe DILI patients with hyperbilirubinemia (TBil >243 µmol/L) (79). GCs combined with ursodesoxycholic acid appears to be safe, and leads to a more rapid reduction in bilirubin and transaminases after severe DILI (80). However, opposite result was found by Wan et al. that prednisone was not beneficial for the treatment of severe DILI (81). Heretofore, among the several liver diseases, AIH is the most reliable clinical indication for GC treatment (82). In patients with suspected drug induced AIH who are receiving GCs therapy, withdrawal of treatment once the liver injury has resolved should be followed by careful monitoring (83). Antiepileptic drug-induced liver injury is commonly related with hypersensitivity symptoms and may respond to GCs treatment (84). GCs should be administered in patients with severe alcoholic hepatitis (AH) if there are no contraindications (85). Overall, drug-induced liver failure needs evidence of immunopathogenicity to restore the condition through GCs blocking immune responses.

## 4 The Application Strategy of GC Treatment in Liver Failure

### 4.1 The Dose and Timing of GC Treatment in Liver Failure

There is currently no consensus on the type and dosage of GCs used in LF. GC dose is generally controlled in methylprednisolone (1∼2mg/kg/d) according to current clinical studies. Kotoh et al. investigated the possibility of using high dose of GCs to treat LF. 17 ALF patients underwent three days treatment of 1000mg methylprednisolone daily, and 13 of them were cured without serious complications, two died, and two received LT (64). The relevance of high-dose GCs in the treatment of severe acute exacerbation of CHB and the early stage of ALF was explored by other researchers. They indicated a slim advantage in terms of survival and liver regeneration in the GC treated group, but there was no significant difference, whereas patients with a poor basic condition and advanced liver damage at the start of treatment had a poor prognosis (50, 65). It will not function if a high dose of GCs are given during LF due to a decrease in the number of GC receptors on the surface of cells in the liver tissue, and there may also be an increase in the likelihood of GC adverse effects because GCs have the potential to cause substantial liver damage (65, 86, 87). As a result, high-dose GCs are more usually used in ALF patients compared to ACLF patients. And not indicated for individuals particularly with poor basic condition. Low and medium doses are generally used. Currently, some researchers utilize 10 mg dexamethasone once a day for three days to treat patients with HBV-related ACLF. The results revealed that early in the course of a severe acute exacerbation of chronic hepatitis B, combined with standard treatment, low-dose, short-term glucocorticoid treatment dramatically decreased the probability of progression to liver failure and shortened hospitalization time, without raising the complication rate (88). Low doses of GCs primarily depress cellular immunity, but high doses of GCs lower humoral immunity by suppressing B cells and antibody generation (89). However, there are certain variances in the dosage of GC used for various reasons. Prednisone 40 mg/d, according to some research, can be taken early in alcohol induced LF (90). When AIH is induced to LF, an initial dose of 20-50 mg/d methylprednisolone is used to provide a stronger curative effect (91). Some studies employed 1.5 mg/kg/d as the beginning dose for CHB-related LF and eventually reached excellent outcomes after progressively lowering the dose according to the disease (92).

When the effectiveness of GC treatment cannot be established in a clinical setting, the concept of safety requires that any potential adverse effects of GCs be maintained within a manageable range. GCs can considerably lower the number of lymphocytes in circulation by inhibiting the presence of phagocytic cells to the antigen, promoting the destruction and disintegration of lymphocytes, and developing the removal of lymphocytes from blood vessels (93). Even though that GCs can raise the risk of infection and upper gastrointestinal bleeding, as well as other complications, their adverse effects are manageable. As a result, it is critical to screen for and monitor adverse effects in individuals with liver failure who are taking GCs.

GC intervention in the early stages of LF has been demonstrated in several studies to improve prognosis and minimize death (63). Zhao et al. believed that GCs should be utilized when the MELD score is less than 35, the HE score is less than 4, and the ALT level is ≥ 30 ULN (14). When the MELD score is less than 27 and hepatic encephalopathy is less than stage II in AIH-related LF, the benefit of GCs is greatest (94). However, until recently, there were no clear quantitative indicators for GC therapy in LF, we believe that the age, basic conditions, and complications of the patients should all be considered. As a result, more clinical experience should be very important for doctors.

### 4.2 Problems of GCs Application in Liver Failure

#### 4.2.1 GC Resistance

Some ACLF patients have a low sensitivity to GCs treatment (95). GCs *via* binding to their intracellular receptor (GR) to have powerful anti-inflammatory activities. Decreased GR in many inflammatory diseases confers GC resistance (GCR) and undermines glucocorticoid therapy efficacy. GCR is clearly acquired through persistent inflammatory injury (96–98). Tjandra et al. reported a significant decrease in hepatic T lymphocyte GR mRNA and protein levels in experimental cholangitis rats, demonstrating that hepatic T cell resistance to increased cortisol levels is at least partially mediated by decreased GR expression (99). In AIH patients, GR expression in peripheral mononuclear cells was shown to be closely related with GCR and to impact the outcome of therapy and the degree of disease severity (100, 101). Moreover, there is a dynamic process in the immune state of LF. It is reported that inactivation of functional T cells is a key step in the progression of systemic immunological dysfunction in ALF (102). And proinflammatory cytokines are involved in the pathogenesis of ALF (103). Further studies reported that the serum cortisol level and the percentage of GR+T lymphocytes were significantly decreased in HBV-ACLF patients compared with CHB patients and healthy controls. The relative GR alpha mRNA expression was significant decreased in ACLF patients (95). Recently, Wang et al. noted that in ACLF patients, GR alpha expression was negatively regulated by miR-124a. MicroRNA-124a contributes to GCR in ACLF by negatively regulating GR alpha (104).

#### 4.2.2 Side Effects of GC Therapy in Liver Failure

The immune system is depressed during GC therapy, which raises the risk of secondary infection and the spread and aggravation of the primary illness, as well as the chance of systemic infection and sepsis (14, 66, 105). According to the CANONIC research of the EASL chronic liver failure (EASL-CLIF) Alliance, almost a third of patients with ACLF will be complicated with bacterial infection. Similarly, the EASL-CLIF and the North American Federation of End-stage Liver Diseases (NACSELD) have discovered that some individuals with liver cirrhosis develop ACLF due to coinfection (106, 107). When LF strikes, the intestinal barrier weakens and microecological changes occur, allowing intestinal flora to migrate and endotoxin to enter the bloodstream, resulting in infection (108). At the same time, microorganisms increase the risk of infection after avoiding the immune system and entering the circulation owing to immune escape and immunological paralysis during LF (109). Furthermore, genetic variables have a role in raising the likelihood of coinfection in LF patients (110, 111).

Sepsis is a common complication of ACLF (112), GCs have been tested and widely used in sepsis patients (113). Although it is an acute systemic inflammatory disease, GCs are hardly useful in sepsis (114). One reason to explain the rather poor successes of GCs in sepsis is that a profound GCR has developed. GCR has already developed by the time sepsis are diagnosed and treated. Many researchers have described GCR in cohorts of sepsis patients. Levels of GR mRNA in peripheral mononuclear cells (PBMCs) were found reduced in sepsis children (115). And Dekelbab et al. reported reduced GR protein levels in some organs (such as liver, brain, muscle) during sepsis (116). An increased expression of miR124 was associated with reduced GR expression in T cells of sepsis patients was also reported (117). Furthermore, Guerrero et al. found a temporary increase of the dominant negative GR beta in PBMCs during sepsis (118). Sepsis is associated with GCR significantly, which might be due to a decrease in GR expression or response.

GCs can also suppress gastric mucus secretion while increasing stomach acid and pepsin secretion, resulting in ulcers, perforations, and gastrointestinal (GI) bleeding. As LF is associated with a high risk of severe GI bleeding, using GCs in LF will significantly increase the risk of causes. Although the specific mechanism by which GCs may cause GI bleeding is unknown, GCs may inhibit tissue repair, thus causing delayed wound healing (119). Furthermore, aberrant blood pressure may emerge because of the pharmacological properties of GCs, as well as electrolyte and blood glucose abnormalities, potentially increasing the risk of hepatic encephalopathy, hepatorenal syndrome, and other consequences in LF.

### 4.3 Strategies of GCs Application in Liver Failure

Secondary infection is one of the most serious concerns associated with the use of GCs in LF, posing a secondary threat to the prognosis. Antibiotic prophylaxis can avoid LF consequences including peritonitis and upper gastrointestinal bleeding (120). Currently, it is generally recommended to choose quinolones for prophylactic anti-infective treatment (121). Simultaneously, by increasing gut flora, stimulating gastrointestinal peristalsis, managing autoimmunity, and strengthening nursing care, we can lower the chance of infection (122–124). Opportunistic fungal infections have emerged as a major cause of morbidity and mortality in immunocompromised patients including those who have received GC treatment (125). Although some studies have found that the prognosis of ACLF patients with fungal infection does not improve after active antifungal therapy, since all survivors have received antifungal therapy, it is still recommended to begin antifungal therapy as soon as possible in the early detection of fungal infection (126).

GI bleeding is common in LF patients, especially in ACLF patients with esophageal varices. The current GI bleeding in ALF patients is 1.5% (127). Patients who were taking high-dose glucocorticoids alone had a slight increased relative risk for developing GI bleeding (128). Pharmacologic suppression of stomach acid secretion has been proven to prevent GI bleeding (129, 130). Proton pump inhibitors (PPIs) are effective method to prevent peptic ulcer disease and GI bleeding in ALF (131). It is reported that in drug-induced liver injury, a PPI might be useful to prevent GI bleeding when GCs used (132). A study of HBV-related LF showed that GC therapy accompanied by prophylactic medication with PPI can prevent the severe side effects of GI bleeding of GC therapy (92). As a result, while using GCs in LF patients, more attention must be taken, and the stomach mucosa should be actively preserved to avoid GI bleeding.

Because of the immunosuppression caused by using GCs, LF patients with basic viral hepatitis may activate the virus. HBsAg positive LF patients should start antiviral therapy as soon as possible to inhibit virus replication. GCs can cause feelings of euphoria, excitation, sleeplessness, and even severe mental problems including hallucinations and insanity. It should be given special attention to patients’ mental states. Medication should be discontinued as soon as significant mental problems are discovered. Patients taking GCs for a long time will develop osteoporosis, timely calcium supplement will be useful. Since the proposal of GCs in the treatment of LF has been controversial, its strong immunosuppressive effect and significant efficacy not only bring great temptation to us but also make us face the risk of serious adverse reactions.

## 5 Conclusions

Although the notion of administering GCs to treat LF has circulated for a long time, no conclusive evidence has been provided of its therapeutic efficacy. Some data was from non-randomized studies or carried out in small groups (62, 64, 67). Here, we summarized several published articles referred GC treatment in different status of LF in **Table 1**.

**Table 1:** GC treatment in different etiology of liver failure.

Reference	Year	Nation	Etiology	Type/Stage	Numbers of patients (total/GC treatment)	Intervention	Duration of therapy	Outcome
Gregory et al. (6)	1976	USA	HBV	ALF	29/14	Methylprednisolone (32–40 mg/day)	2 weeks	Do not improve prognosis
Greenber et al. (7)	1981	USA	HBV	ALF	16/8	Methylprednisolone (48 mg/day)	4 weeks	Do not improve prognosis
Ware et al. (8)	1981	USA	HBV	PLF	77/37	Prednisone (40 mg/day)	1 week	Do not improve prognosis and liver function
Rakela et al. (9)	1991	USA and Canada	Drug/hepatitis virus(A/B)	ALF	64/46	Hydrocortisone (400-800 mg/day)	Within 38 months	Do not improve prognosis
Kotoh et al. (64)	2006	Japan	HBV	ALF	34/17	Methylprednisolone (1000mg/day)	3 days	Prevent the ALF progression
Ichai et al. (69)	2007	France	AIH	ALF	16/8	Prednisone (1 mg/kg/d)	median of 2.5 days	Little benefit in severe and fulminant forms of AIH
Fujiwara et al. (50)	2010	Japan	HBV	ALF	10/10	Methylprednisolone (1000mg/day) or Prednisone (40-60 mg/day)	21-183 days	Required in the early stage
Zhang et al. (62)	2011	China	HBV	PLF	170/56	Dexamethasone (10 mg/day)	5 days	Improve prognosis and liver function
Wree et al. (80)	2011	UK	Drug	ALF	15/6	Prednisone (low dose)	3 days	Improve liver function (combined with ursodesoxycholic acid)
Zhao et al. (59)	2012	China	HBV	ACLF	56/30	Methylprednisolone (80 mg/day)	3 days	Improve prognosis
Karkhanis et al. (70)	2014	USA	HBV	ALF	361/62	Prednisone (40-60mg/d)	24-32.5 days	Do not improve prognosis
Zhu et al. (91)	2014	China	AIH	LF	22/7	Prednisolone (20-50 mg/d)	16-105 days	Improve prognosis
Fujiwara et al. (65)	2014	Japan	HBV	ALF	31/9	Methylprednisolone (1000mg/d)	3 days	Improved prognosis and liver regeneration
Chen et al. (61)	2014	China	HBV	ACLF	134/31	Dexamethasone (10 mg/d/person)	3 days	Do not improve prognosis and liver function
Zhao et al. (14)	2016	China	HBV	ALF SALF	73/34 165/21	Dexamethasone (5-30mg/d)	1-10 days	Improve prognosis
Yasui et al. (66)	2016	Japan	HBV	ALF	110/78	methylprednisolone (1,000 mg/d)	3 days	Do not increase the incidence of infection
Hu et al. (79)	2016	China	Drug	ALF	203/53	Methylprednisolone(60-120mg/d) or prednisolone (40-60mg/d)	3-5 days	Improve prognosis
Fujiwara et al. (67)	2018	Japan	HBV	ALF	19/14	Methylprednisolone (1,000 mg/d) or prednisolone (60 mg/d)	according to the response	Improve liver function (Combined with nucleoside analogs)
Anastasiou et al. (72)	2018	Germany	AIH	ALF	125/32	prednisone or prednisolone (60-500mg/d)	Not mention	Improve prognosis
Huang et al. (15)	2019	China	HBV	ACLF	293/162	Prednisone (1mg/kg) or methylprednisolone or dexamethasone	within 1 week	Do not improve prognosis (transplant free patients)
Zachou et al. (74)	2019	Greece	AIH	ALF	184/34	Prednisolone(1000mg/d)	3 days	Safe and efficient
Wan et al. (81)	2019	China	Drug	ALF	90/66	Prednisone (median 40mg/d)	7-86 days (median 21.5days)	Safe but not beneficial
Jia et al. (27)	2020	China	HBV	ACLF	171/83	methylprednisolone (1.5 mg/kg/day [day 1–3], 1 mg/kg/day [day 4–5], and 0.5 mg/kg/day [day 6–7])	7 days	Improve prognosis
Wu et al. (88)	2021	China	HBV	PLF	125/62	Dexamethasone (10mg/day)	3 days	Reduce the risk of progression
Xu et al. (58)	2021	China	HBV	ACLF	349/155	Methylprednisolone or Prednisone	28 days	Do not improve liver function, but improve prognosis

ALF, acute liver failure; PLF, preliver failure; ACLF, acute-on-chronic liver failure; LF, liver failure; SALF, sub-acute liver fail.

Given all of that, due to the intricate pathophysiology of LF, it is critical to investigate immunological manifestations with various etiologies. In the treatment of LF, we should personalize each patient’s treatment plan, prioritize patient safety, monitor, and avoid any adverse responses to GCs in a timely manner, all of which will help patients obtain more benefit and improve their prognosis. To provide clinical professionals with a suitable treatment plan based on evidence-based medicine, further larger randomized clinical trials are required.

## Author Contributions

CY wrote this manuscript, WL designed this manuscript, LL and KZ provided literatures review. All authors contributed to the article and approved the submitted version.

## Funding

Provincial natural science foundation of Anhui (1908085QH331).

## Conflict of Interest

The authors declare that the research was conducted in the absence of any commercial or financial relationships that could be construed as a potential conflict of interest.

## Publisher’s Note

All claims expressed in this article are solely those of the authors and do not necessarily represent those of their affiliated organizations, or those of the publisher, the editors and the reviewers. Any product that may be evaluated in this article, or claim that may be made by its manufacturer, is not guaranteed or endorsed by the publisher.
